# Construction and validation of a nomogram for predicting prolonged air leak after minimally invasive pulmonary resection

**DOI:** 10.1186/s12957-022-02716-w

**Published:** 2022-08-03

**Authors:** Rongyang Li, Mengchao Xue, Zheng Ma, Chenghao Qu, Kun Wang, Yu Zhang, Weiming Yue, Huiying Zhang, Hui Tian

**Affiliations:** Department of Thoracic Surgery, Qilu Hospital of Shandong University, Jinan, 250000 Shandong China

**Keywords:** Prolonged air leak, Risk factor, Minimally invasive pulmonary resection, Predictive model, Nomogram

## Abstract

**Background:**

Prolonged air leak (PAL) remains one of the most frequent postoperative complications after pulmonary resection. This study aimed to develop a predictive nomogram to estimate the risk of PAL for individual patients after minimally invasive pulmonary resection.

**Methods:**

Patients who underwent minimally invasive pulmonary resection for either benign or malignant lung tumors between January 2020 and December 2021 were included. All eligible patients were randomly assigned to the training cohort or validation cohort at a 3:1 ratio. Univariate and multivariate logistic regression were performed to identify independent risk factors. All independent risk factors were incorporated to establish a predictive model and nomogram, and a web-based dynamic nomogram was then built based on the logistic regression model. Nomogram discrimination was assessed using the receiver operating characteristic (ROC) curve. The calibration power was evaluated using the Hosmer-Lemeshow test and calibration curves. The nomogram was also evaluated for clinical utility by the decision curve analysis (DCA).

**Results:**

A total of 2213 patients were finally enrolled in this study, among whom, 341 cases (15.4%) were confirmed to have PAL. The following eight independent risk factors were identified through logistic regression: age, body mass index (BMI), smoking history, percentage of the predicted value for forced expiratory volume in 1 second (FEV1% predicted), surgical procedure, surgical range, operation side, operation duration. The area under the ROC curve (AUC) was 0.7315 [95% confidence interval (CI): 0.6979–0.7651] for the training cohort and 0.7325 (95% CI: 0.6743–0.7906) for the validation cohort. The *P* values of the Hosmer-Lemeshow test were 0.388 and 0.577 for the training and validation cohorts, respectively, with well-fitted calibration curves. The DCA demonstrated that the nomogram was clinically useful. An operation interface on a web page (https://lirongyangql.shinyapps.io/PAL_DynNom/) was built to improve the clinical utility of the nomogram.

**Conclusion:**

The nomogram achieved good predictive performance for PAL after minimally invasive pulmonary resection. Patients at high risk of PAL could be identified using this nomogram, and thus some preventive measures could be adopted in advance.

**Supplementary Information:**

The online version contains supplementary material available at 10.1186/s12957-022-02716-w.

## Background

With the popularization of low-dose computed tomography screening, small pulmonary nodules have been increasingly detected in recent years [1]. Minimally invasive pulmonary resection, including robotic-assisted thoracic surgery (RATS) and video-assisted thoracic surgery (VATS), has replaced conventional thoracotomy as the dominant surgical treatment for pulmonary nodules with satisfactory outcomes [2, 3]. Prolonged air leak (PAL) remains one of the most frequent postoperative complications after pulmonary resection, with a reported incidence of up to 30% [4–6]. PAL is closely associated with increased pain from longer duration of chest tubes, prolonged hospital length of stay, higher financial cost, increased risk of some complications, and poor postoperative quality of life [7–9]. Although many methods for PAL management, such as fibrin sealants, pleural tents, pleurodesis, digital chest drainage system, and endobronchial valves, have been proven effective, PAL remains a great challenge for thoracic surgeons [4, 10]. Therefore, identification of patients at high risk for PAL could enable the doctors to take additional effective procedures to prevent its occurrence in advance.

At present, many risk factors have been identified to increase the incidence of postoperative PAL, including male sex, low body mass index (BMI), smoking history, reduced percentage of the predicted value for forced expiratory volume in 1 second (FEV1% predicted), previous chemoradiotherapy, presence of diabetes and lobectomy [5, 6, 11]. Although a few predictive models for stratifying patients with high risk for PAL have been developed in recent years [12–18], there is still no standard model to estimate the incidence of PAL. The majority of the previously reported models were based on the data from European and the USA, which might make it not applicable for Asian populations [12–14, 16–18]. In addition, the differences in PAL definitions and patient selection criteria made the results of these studies less credible [12–15, 18]. Moreover, in the era of minimally invasive pulmonary resection as the dominant surgical procedure, traditional thoracotomy is rarely performed, and thus the clinical utility of previous models incorporating thoracotomy as a risk factor would be greatly reduced.

The purpose of this study is to develop a clinical prediction model and nomogram to estimate the risk of PAL after minimally invasive pulmonary resection for both benign and malignant lung tumors using preoperative and intraoperative characteristics in a large cohort from a single center in China. The predictive nomogram could stratify the patients into different risk categories and assist thoracic surgeons in making clinical decision.

## Patients and methods

This retrospective study was approved by the Ethics Committee of the Qilu Hospital of Shandong University (registration number: KYLL-202008-023-1) and all patients provided informed consent for the use of their clinical information.

### Patients selection

A prospectively maintained departmental database of the Qilu Hospital of Shandong University was retrieved for patients who underwent minimally invasive pulmonary resection from January 2020 to December 2021. The exclusion criteria were: (I) patients aged < 18 years old; (II) lung volume reduction surgery and bulla resection; (III) pneumonectomy; (IV) pulmonary resection with mediastinal mass resection; (V) thoracotomy; and (VI) incomplete perioperative data. All enrolled patients were randomly assigned to the training cohort or validation cohort at a 3:1 ratio using a random split-sample method. The training cohort was used to develop the predictive nomogram, while the validation cohort was used to verify the performance of the nomogram.

### Data collection and variable definitions

The following data of eligible patients were collected from the database of Qilu Hospital: (I) demographic data: age, gender, BMI, smoking history, preoperative comorbidities [hypertension, diabetes mellitus, and chronic obstructive pulmonary diseases (COPD)], neoadjuvant therapy, and history of thoracic surgery; (II) preoperative evaluation data: FEV1% predicted, percentage of the predicted value of maximal voluntary ventilation (MVV% predicted), American Society of Anesthesiologists (ASA) score, ABO blood type, peripheral blood lymphocyte count, albumin, and prognostic nutritional index (PNI); (III) surgical data: surgical technique (RATS or VATS), surgical procedure (sublobar resection or lobectomy), surgical range (mono-lobe or multi-lobe), operation side, operation duration, total number of dissected lymph nodes (LNs), and tumor size.

PAL was defined as air leakage more than 5 consecutive days after surgery. A conventional drainage system was used for the majority of the cases, and a digital continuous negative pressure drainage device with a negative pressure range of 6-10 cm water column was applied for patients with significant PAL. The chest tube could be removed if there was no pneumonia, subcutaneous emphysema or pneumothorax with a daily drainage of less than 200ml. PNI was calculated using the following formula: PNI (%) = albumin (g/L) + 5 × lymphocyte (10^9^/L). Tumor size was defined as the maximum tumor diameter.

### Construction of the PAL nomogram

Univariate logistic regression analysis was performed to identify potential risk factors for PAL. All factors with a *P* value less than 0.20 in univariate analysis were included in further multivariate logistic regression analysis. Independent risk factors (*P* <0.05 in multivariate logistic regression) were finally used to develop the predictive model. A nomogram was then constructed based on the results of a multivariate logistic regression model by using the “rms” and “DynNom” packages in R project software (version 4.1.1; http://www.r-project.org/). A score of each variable was calculated using the regression model, and the predicted probability of PAL could be derived by summing the scores of the individual variables.

### Nomogram performance

The performance of the predictive nomogram was assessed by discrimination, calibration, and clinical utility. Discrimination is the capacity that a model can correctly distinguish between events and non-events. We used the receiver operating characteristic (ROC) curve to evaluate the discrimination efficiency of the predictive nomogram [19]. Calibration measures how closely the predicted probabilities are consistent with the actual outcomes. The Hosmer-Lemeshow test was used to evaluate the calibration power, and a *P* value larger than 0.05 indicated satisfactory calibration [20]. A nomogram calibration plot was then formed to further evaluate calibration. Internal validation was performed by using the bootstrapping method with 1000 repetitions [21]. Decision curve analysis (DCA) was performed to evaluate the clinical utility of the predictive nomogram based on net benefits at different threshold probabilities [22]. The optimal cutoff value was determined based on the ROC curve analysis of the training cohort when the Youden index (sensitivity + specificity − 1) reached the maximum value.

### Statistical analysis

Categorical variables were compared using the Pearson Chi-squared test or Fisher exact test. Normally distributed continuous variable was presented as mean ± standard deviation (SD), and the Student *t*-test was used for comparison. For continuous variables that are not normally distributed, the data was presented as median (interquartile range [IQR]) and compared by the Mann–Whitney *U* test between the groups. A two-sided *P* value < 0.05 was considered statistically significant. R Project software (v4.1.1; http://www.R-project.org) and SPSS software (v25.0; SPSS Inc., Chicago, IL, USA) were used for data analysis.

## Results

### Patient characteristics

**Fig. 1:**
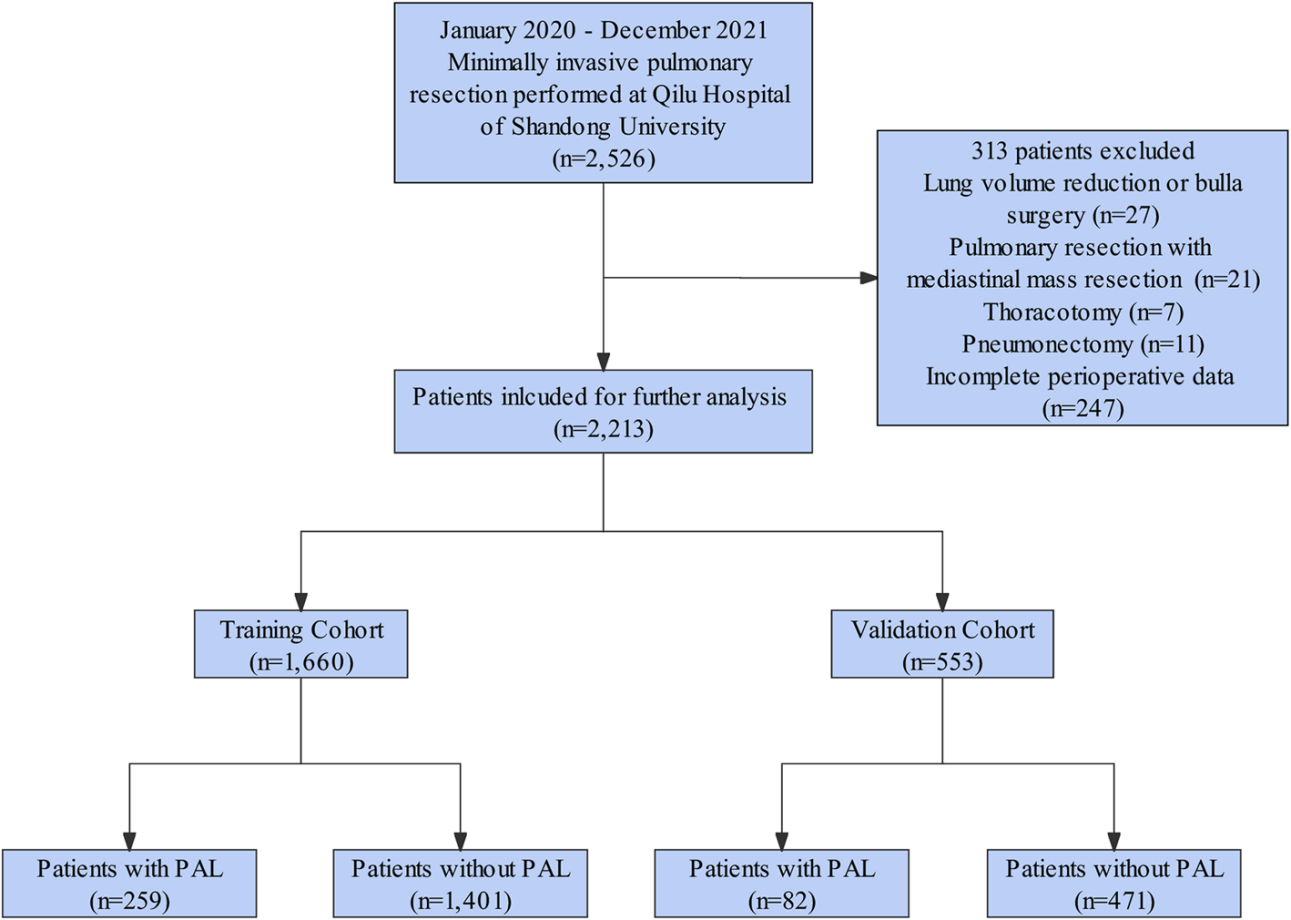
Flow diagram of patient selection through the study. PAL, prolonged air leak

The procedure of identification and selection of the eligible patients is illustrated in Fig. 1. Finally, a total of 2213 eligible patients were included in our study, among whom, the incidence of PAL was 15.4% (341/2213). The enrolled patients were then randomly assigned to the training cohort (*n* = 1660) or validation cohort (*n* = 553) at a 3:1 ratio, and there were no significant differences in all variables between the two cohorts (Table 1). According to the presence or absence of PAL, patients were divided into PAL and non-PAL groups. The characteristics of both groups in the training and validation cohorts are presented in Table 2.

**Table 1:** Patients’ characteristics of the training cohort and validation cohort

Characteristics	All cohort (***n*** = 2213)	Training cohort (***n*** = 1660)	Validation cohort (***n*** = 553)	***P*** ^**†**^
Age (years), median (IQR)	58.00 (51.00–66.00)	58.00 (51.00–66.00)	58.00 (52.00–66.00)	0.607
Gender, *n* (%)				0.353
Female	1274 (57.6)	965 (58.1)	309 (55.9)	
Male	939 (42.4)	695 (41.9)	244 (44.1)	
BMI (kg/m^2^), median (IQR)	24.77 (22.77–27.01)	24.71 (22.77–26.95)	25.04 (22.83–27.26)	0.153
Smoking history, *n* (%)				0.314
Non-smoker	1625 (73.4)	1228 (74.0)	397 (71.8)	
Smoker	588 (26.6)	432 (26.0)	156 (28.2)	
Hypertension, *n* (%)				0.248
No	1591 (71.9)	1204 (72.5)	387 (70.0)	
Yes	622 (28.1)	456 (27.5)	166 (30.0)	
Diabetes mellitus, *n* (%)				0.156
No	1926 (87.0)	1435 (86.4)	491 (88.8)	
Yes	287 (13.0)	225 (13.6)	62 (11.2)	
COPD, *n* (%)				0.804
No	2191 (99.0)	1644 (99.0)	547 (98.9)	
Yes	22 (1.0)	16 (1.0)	6 (1.1)	
Neoadjuvant therapy, *n* (%)				0.640
No	2174 (98.2)	1632 (98.3)	542 (98.0)	
Yes	39 (1.8)	28 (1.7)	11 (2.0)	
History of thoracic surgery, *n* (%)				0.255
No	2187 (98.8)	1638 (98.7)	549 (99.3)	
Yes	26 (1.2)	22 (1.3)	4 (0.7)	
FEV1 % predicted (%), median (IQR)	104.46 (93.35–115.58)	104.55 (92.89–115.71)	104.35 (94.63–115.11)	0.674
MVV % predicted (%), median (IQR)	103.15 (90.24–115.63)	102.74 (89.91–115.46)	104.82 (91.18–116.26)	0.201
Blood type, *n* (%)				0.670
A	637 (28.8)	479 (28.9)	158 (28.6)	
B	763 (34.5)	578 (34.8)	185 (33.5)	
AB	230 (10.4)	165 (9.9)	65 (11.8)	
O	583 (26.3)	438 (26.4)	145 (26.2)	
Lymphocyte (×10^9^/L), median (IQR)	1.78 (1.46–2.18)	1.79 (1.45–2.18)	1.77 (1.49–2.16)	0.791
Albumin (g/L), median (IQR)	59.70 (57.60–61.90)	59.80 (57.60–61.90)	59.70 (57.50–61.90)	0.635
PNI (%), median (IQR)	68.95 (66.05–71.65)	68.95 (66.05–71.60)	68.90 (65.85–71.98)	0.839
ASA score, *n* (%)				0.185
I	212 (9.6)	162 (9.8)	50 (9.0)	
II	1934 (87.4)	1454 (87.6)	480 (86.8)	
III	67 (3.0)	44 (2.7)	23 (4.2)	
Surgical technique, *n* (%)				0.200
RATS	743 (33.6)	545 (32.8)	198 (35.8)	
VATS	1470 (66.4)	1115 (67.2)	355 (64.2)	
Surgical procedure, *n* (%)				0.932
Sublobar resection	1041 (47.0)	780 (47.0)	261 (47.2)	
Lobectomy	1172 (53.0)	880 (53.0)	292 (52.8)	
Surgical range, *n* (%)				0.256
Mono-lobe	1909 (86.3)	1424 (85.8)	485 (87.7)	
Multi-lobe	304 (13.7)	236 (14.2)	68 (12.3)	
Operation side, *n* (%)				0.972
Left-sided	875 (39.5)	656 (39.5)	219 (39.6)	
Right-sided	1338 (60.5)	1004 (60.5)	334 (60.4)	
Operation duration (min), median (IQR)	90.00 (70.00–120.00)	90.00 (70.00–120.00)	90.00 (70.00–115.00)	0.181
Number of LNs dissected, median (IQR)	6.00 (4.00–10.00)	6.00 (4.00–10.00)	6.00 (4.00–10.00)	0.941
Tumor size (cm), median (IQR)	1.40 (1.00–2.00)	1.40 (1.00–2.00)	1.50 (1.00–2.00)	0.885
Prolonged air leak				0.662
No	1872 (84.6)	1401 (84.4)	471 (85.2)	
Yes	341 (15.4)	259 (15.6)	82 (14.8)	

*IQR* interquartile range, *BMI* body mass index, *COPD* chronic obstructive pulmonary diseases, *FEV1* forced expiratory volume in one second, *MVV* maximal voluntary ventilation, *PNI* prognostic nutritional index, *ASA* American Society of Anesthesiologists, *RATS* robotic-assisted thoracic surgery, *VATS* video-assisted thoracic surgery, *LN* lymph node

^†^ *P*-value for the comparison between training cohort and validation cohort

**Table 2:** Clinical characteristics of patients with or without PAL in training cohort and validation cohort

Characteristics	Training cohort			Validation cohort		
	Non-PAL (***n*** = 1401)	PAL (***n*** = 259)	***P***	Non-PAL (***n*** = 471)	PAL (***n*** = 82)	***P***
Age (years), median (IQR)	58.0 (51.0–65.0)	62.0 (54.0–69.0)	<0.001	57.0 (51.0–65.0)	63.0 (55.5–69.0)	0.002
Gender, *n* (%)			<0.001			<0.001
Female	842 (60.1)	123 (47.5)		284 (60.3)	25 (30.5)	
Male	559 (39.9)	136 (52.5)		187 (39.7)	57 (69.5)	
BMI (kg/m^2^), median (IQR)	24.9 (22.9–27.1)	24.0 (22.1–26.0)	<0.001	25.3 (23.0–27.3)	24.1 (22.3–26.6)	0.052
Smoking history, *n* (%)			<0.001			0.009
Non-smoker	1074 (76.7)	154 (59.5)		348 (73.9)	49 (59.8)	
Smoker	327 (23.3)	105 (40.5)		123 (26.1)	33 (40.2)	
Hypertension, *n* (%)			0.299			0.872
No	1023 (73.0)	181 (69.9)		329 (69.9)	58 (70.7)	
Yes	378 (27.0)	78 (30.1)		142 (30.1)	24 (29.3)	
Diabetes mellitus, *n* (%)			0.983			0.760
No	1211 (86.4)	224 (86.5)		419 (89.0)	72 (87.8)	
Yes	190 (13.6)	35 (13.5)		52 (11.0)	10 (12.2)	
COPD, *n* (%)			0.028			0.219
No	1391 (99.3)	253 (97.7)		467 (99.2)	80 (97.6)	
Yes	10 (0.7)	6 (2.3)		4 (0.8)	2 (2.4)	
Neoadjuvant therapy, *n* (%)			0.066			0.383
No	1381 (98.6)	251 (96.9)		460 (97.7)	82 (100.0)	
Yes	20 (1.4)	8 (3.1)		11 (2.3)	0	
History of thoracic surgery, *n* (%)			0.560			0.107
No	1381 (98.6)	257 (99.2)		469 (99.6)	80 (97.6)	
Yes	20 (1.4)	2 (0.8)		2 (0.4)	2 (2.4)	
FEV1 % predicted (%), median (IQR)	105.3 (93.9–116.0)	100.5 (87.1–113.2)	<0.001	105.3 (95.4–115.8)	99.2 (86.9–111.4)	<0.001
MVV % predicted (%), median (IQR)	103.2 (91.1–116.0)	98.2 (85.1–112.1)	<0.001	104.9 (92.5–117.1)	95.2 (85.7–107.3)	<0.001
Blood type, *n* (%)			0.188			0.199
A	396 (28.3)	83 (32.0)		140 (29.7)	18 (22.0)	
B	490 (35.0)	88 (34.0)		151 (32.1)	34 (41.5)	
AB	148 (10.6)	17 (6.6)		53 (11.3)	12 (14.6)	
O	367 (26.2)	71 (27.4)		127 (27.0)	18 (22.0)	
Lymphocyte (×10^9^/L), median (IQR)	1.79 (1.46–2.20)	1.73 (1.41–2.13)	0.075	1.78 (1.49–2.16)	1.68 (1.43–2.20)	0.306
Albumin (g/L), median (IQR)	59.8 (57.6–61.9)	59.9 (57.6–62.2)	0.395	59.7 (57.4–61.9)	60.1 (57.7–62.1)	0.765
PNI (%), median (IQR)	69.0 (66.1–71.6)	68.8 (65.8–72.0)	0.992	69.0 (66.0–72.0)	68.8 (65.0–72.1)	0.840
ASA score, *n* (%)			0.005			0.355
I	143 (10.2)	19 (7.3)		46 (9.8)	4 (4.9)	
II	1228 (87.7)	226 (87.3)		406 (86.2)	74 (90.2)	
III	30 (2.1)	14 (5.4)		19 (4.0)	4 (4.9)	
Surgical technique, *n* (%)			0.316			0.066
RATS	453 (32.3)	92 (35.5)		176 (37.4)	22 (26.8)	
VATS	948 (67.7)	167 (64.5)		295 (62.6)	60 (73.2)	
Surgical procedure, *n* (%)			<0.001			<0.001
Sublobar resection	712 (50.8)	68 (26.3)		239 (50.7)	22 (26.8)	
Lobectomy	689 (49.2)	191 (73.7)		232 (49.3)	60 (73.2)	
Surgical range, *n* (%)			0.001			0.288
Mono-lobe	1219 (87.0)	205 (79.2)		416 (88.3)	69 (84.1)	
Multi-lobe	182 (13.0)	54 (20.8)		55 (11.7)	13 (15.9)	
Operation side, *n* (%)			0.141			0.388
Left-sided	543 (38.8)	113 (43.6)		183 (38.9)	36 (43.9)	
Right-sided	858 (61.2)	146 (56.4)		288 (61.1)	46 (56.1)	
Operation duration (min), median (IQR)	90.0 (70.0–115.0)	110 (85.0–140.0)	<0.001	90.0 (70.0–110.0)	107.5 (80.0–125.0)	<0.001
Number of LNs dissected, median (IQR)	6.0 (4.0–9.0)	8.0 (5.0–12.0)	<0.001	6.0 (4.0–9.0)	7.5 (5.0–12.3)	0.001
Tumor size (cm), median (IQR)	1.3 (0.9–2.0)	1.5 (1.2–2.5)	<0.001	1.3 (0.9–2.0)	2.0 (1.5–3.0)	<0.001

*PAL* prolonged air leak, *IQR* interquartile range, *BMI* body mass index, *COPD* chronic obstructive pulmonary diseases, *FEV1* forced expiratory volume in one second, *MVV* maximal voluntary ventilation, *PNI* prognostic nutritional index, *ASA* American Society of Anesthesiologists, *RATS* robotic-assisted thoracic surgery, *VATS* video-assisted thoracic surgery, *LN* lymph node

### Identification of risk factors for PAL

Univariate and multivariate logistic regression analyses were performed in the training cohort to explore independent risk factors for postoperative PAL (Table 3). Univariate analysis indicated that age, gender, BMI, smoking history, presence of COPD, FEV1% predicted, MVV% predicted, ASA score, surgical procedure, surgical range, operation duration, number of LNs dissected, and tumor size were potential risk factors for PAL (*P* < 0.05). Further multivariate logistic regression revealed that age [odds ratio (OR) = 1.020; 95% confidence interval (CI): 1.004–1.037; *P* = 0.013], BMI (OR = 0.884; 95% CI: 0.842–0.928; *P* < 0.001), Smoking history (OR = 1.628; 95% CI: 1.048–2.531; *P* = 0.030), FEV1% predicted (OR = 0.991; 95% CI: 0.981–1.000; *P* = 0.046), surgical procedure (lobectomy *vs.* sublobar resection; OR = 2.100; 95% CI: 1.424–3.096; *P* < 0.001), surgical range (multi-lobe *vs.* mono-lobe; OR = 1.820; 95% CI: 1.251–2.648; *P* = 0.002), operation side (right *vs.* left; OR = 0.730; 95% CI: 0.545–0.977; *P* = 0.035), and operation duration (OR = 1.009; 95% CI: 1.005–1.012; *P* < 0.001) were independently associated with the occurrence of PAL.

**Table 3:** Univariate and multivariate logistic regression analysis of risk factors for PAL in the training cohort

Variables	PAL rate (%)	Univariate analysis			Multivariate analysis		
		OR	95% CI	***P***	OR	95% CI	***P***
Age	15.6	1.029	1.016–1.043	< 0.001	1.020	1.004–1.037	0.013
Gender				< 0.001			0.819
Female	12.7	Ref.			Ref.		
Male	19.6	1.665	1.276–2.174		0.951	0.619–1.462	
BMI	15.6	0.914	0.875–0.955	< 0.001	0.884	0.842–0.928	< 0.001
Smoking history				< 0.001			0.030
Non-smoker	12.5	Ref.			Ref.		
Smoker	24.3	2.239	1.697–2.955		1.628	1.048–2.531	
Hypertension				0.299			
No	15.0	Ref.					
Yes	17.1	1.166	0.872–1.559				
Diabetes mellitus				0.996			
No	15.6	Ref.					
Yes	15.6	0.996	0.676–1.467				
COPD				0.022			0.080
No	15.4	Ref.			Ref.		
Yes	37.5	3.299	1.188–9.157		2.836	0.883–9.107	
Neoadjuvant therapy				0.063			0.753
No	15.4	Ref.			Ref.		
Yes	28.6	2.201	0.959–5.052		0.862	0.343–2.165	
History of thoracic surgery				0.404			
No	15.7	Ref.					
Yes	9.1	0.537	0.125–2.313				
FEV1 % predicted	15.6	0.983	0.976–0.991	< 0.001	0.991	0.981–1.000	0.046
MVV% predicted	15.6	0.989	0.983–0.996	0.002	0.999	0.994–1.004	0.639
Blood type				0.195			0.166
A	17.3	Ref.			Ref.		
B	15.2	0.857	0.617–1.189		0.877	0.618–1.244	
AB	10.3	0.548	0.315–0.955		0.509	0.282–0.919	
O	16.2	0.923	0.652–1.306		0.912	0.627–1.326	
Lymphocyte	15.6	0.804	0.631–1.023	0.076	0.902	0.697–1.168	0.433
Albumin	15.6	1.015	0.986–1.046	0.313			
PNI	15.6	0.999	0.975–1.024	0.955			
ASA score				0.007			0.264
I	11.7	Ref.			Ref.		
II	15.5	1.385	0.841–2.282		0.879	0.497–1.555	
III	31.8	3.512	1.587–7.775		1.590	0.622–4.063	
Surgical technique				0.316			
RATS	16.9	Ref.					
VATS	15.0	0.867	0.657–1.145				
Surgical procedure				< 0.001			< 0.001
Sublobar resection	8.7	Ref.			Ref.		
Lobectomy	21.7	2.903	2.159–3.902		2.100	1.424–3.096	
Surgical range				0.001			0.002
Mono-lobe	14.4	Ref.			Ref.		
Multi-lobe	22.9	1.764	1.258–2.473		1.820	1.251–2.648	
Operation side				0.141			0.035
Left-sided	17.2	Ref.			Ref.		
Right-sided	14.5	0.818	0.625–1.069		0.730	0.545–0.977	
Operation duration	15.6	1.012	1.009–1.016	< 0.001	1.009	1.005–1.012	< 0.001
Number of LN dissected	15.6	1.076	1.051–1.102	< 0.001	1.019	0.989–1.050	0.224
Tumor size	15.6	1.340	1.210–1.484	< 0.001	1.038	0.921–1.171	0.539

*PAL* prolonged air leak, *BMI* body mass index, *COPD* chronic obstructive pulmonary diseases, *FEV1* forced expiratory volume in one second, *MVV* maximal voluntary ventilation, *PNI* prognostic nutritional index, *ASA* American Society of Anesthesiologists, *RATS* robotic-assisted thoracic surgery, *VATS* video-assisted thoracic surgery, *LN* lymph node

### Nomogram construction

**Fig. 2:**
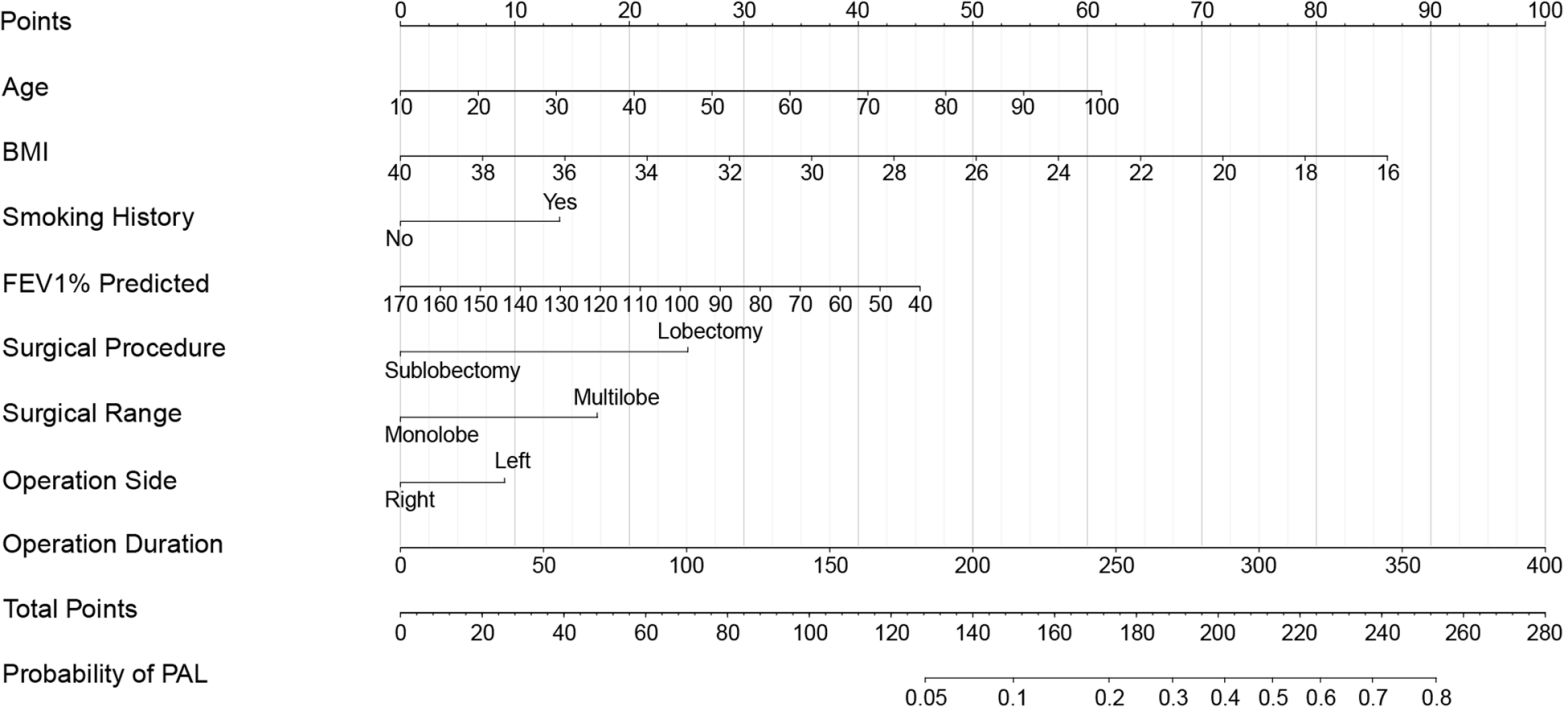
A nomogram for prediction of PAL risk after minimally invasive pulmonary resection. Draw a vertical line from the corresponding axis of each variable to the points axis to acquire the point of this variable. Make a summation of the points for each variable to yield a total score, and the probability of PAL could be estimated by projecting the total score to the lower total point axis. BMI, body mass index; FEV1, forced expiratory volume in one second; PAL, prolonged air leak

All eight independent risk factors for PAL were included to build a logistic regression model. Details of the predictive model are presented in Table 4. Based on the coefficients of the multivariate logistic regression model, a predictive nomogram for PAL was drawn by using the “rms” package of R statistical software (Fig. 2). As shown in this nomogram, there were 11 axes in total, and the axes 2–9 represented the eight variables in the predictive model. The estimated score of each risk factor could be calculated by drawing a perpendicular line to the top points axis, and a further summation could be made to obtain a total point. The total points axis was then used to predict the probability of PAL after minimally invasive pulmonary resection. In addition, to facilitate the wide use of our predictive nomogram for thoracic surgeons, we used the “Dynnom” package to build an operation interface on a web page (https://lirongyangql.shinyapps.io/PAL_DynNom/) to calculate the probability of PAL. By entering or choosing a patient’s preoperative and intraoperative characteristics, the user can obtain the predictive probability of PAL after minimally invasive pulmonary resection.

**Table 4:** Details of the predictive model to calculate the probability of PAL

Risk factors	Coefficient	SE	OR (95% CI)	***P***
Intercept	− 0.276	0.849	0.759	0.745
Age	0.024	0.007	1.024 (1.009–1.038)	0.001
BMI	− 0.125	0.024	0.883 (0.842–0.926)	< 0.001
Smoking history				0.002
Non-smoker	Ref.			
Smoker	0.482	0.158	1.620 (1.188–2.208)	
FEV1% predicted	− 0.012	0.004	0.988 (0.980–0.996)	0.004
Surgical procedure				< 0.001
Sublobectomy	Ref.			
Lobectomy	0.870	0.165	2.388 (1.730–3.296)	
Surgical range				0.002
Mono-lobe	Ref.			
Multi-lobe	0.596	0.188	1.814 (1.255–2.623)	
Operation side				0.032
Left-sided	Ref.			
Right-sided	− 0.315	0.147	0.730 (0.547–0.972)	
Operation duration	0.009	0.002	1.009 (1.005–1.012)	< 0.001

*PAL* prolonged air leak, *BMI* body mass index, *FEV1* forced expiratory volume in one second, *SE* standard error, *OR* odds ratio, *CI* confidence interval. Probability of PAL could be calculated by using the following formula: ln (p/1 − p) = 0.024 × age – 0.125 × BMI + 0.482 × smoking history (non-smoker = 0; smoker = 1) – 0.012 × FEV1% predicted + 0.870 × surgical procedure (sublobectomy = 0; lobectomy = 1) + 0.596 × surgical range (mono-lobe = 0; multi-lobe = 1) – 0.315 × operation side (left = 0; right = 1) + 0.009 × operation duration – 0.276

### Predictive performance and validation of the PAL nomogram

**Fig. 3:**
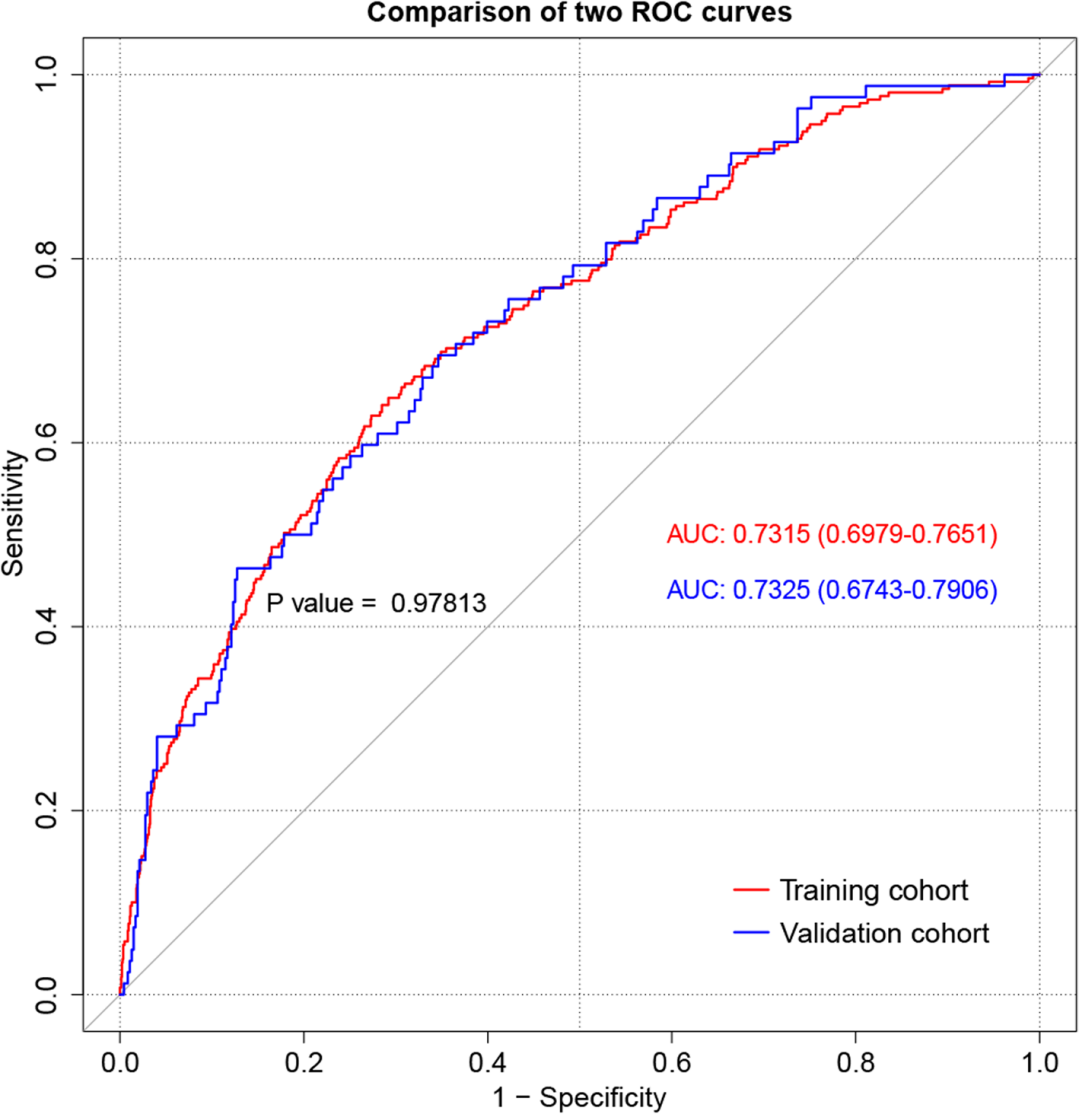
ROC curves of the nomogram for predicting PAL in the training and validation cohorts. ROC, receiver operating characteristic; AUC, areas under the ROC curve; PAL, prolonged air leak

**Fig. 4:**
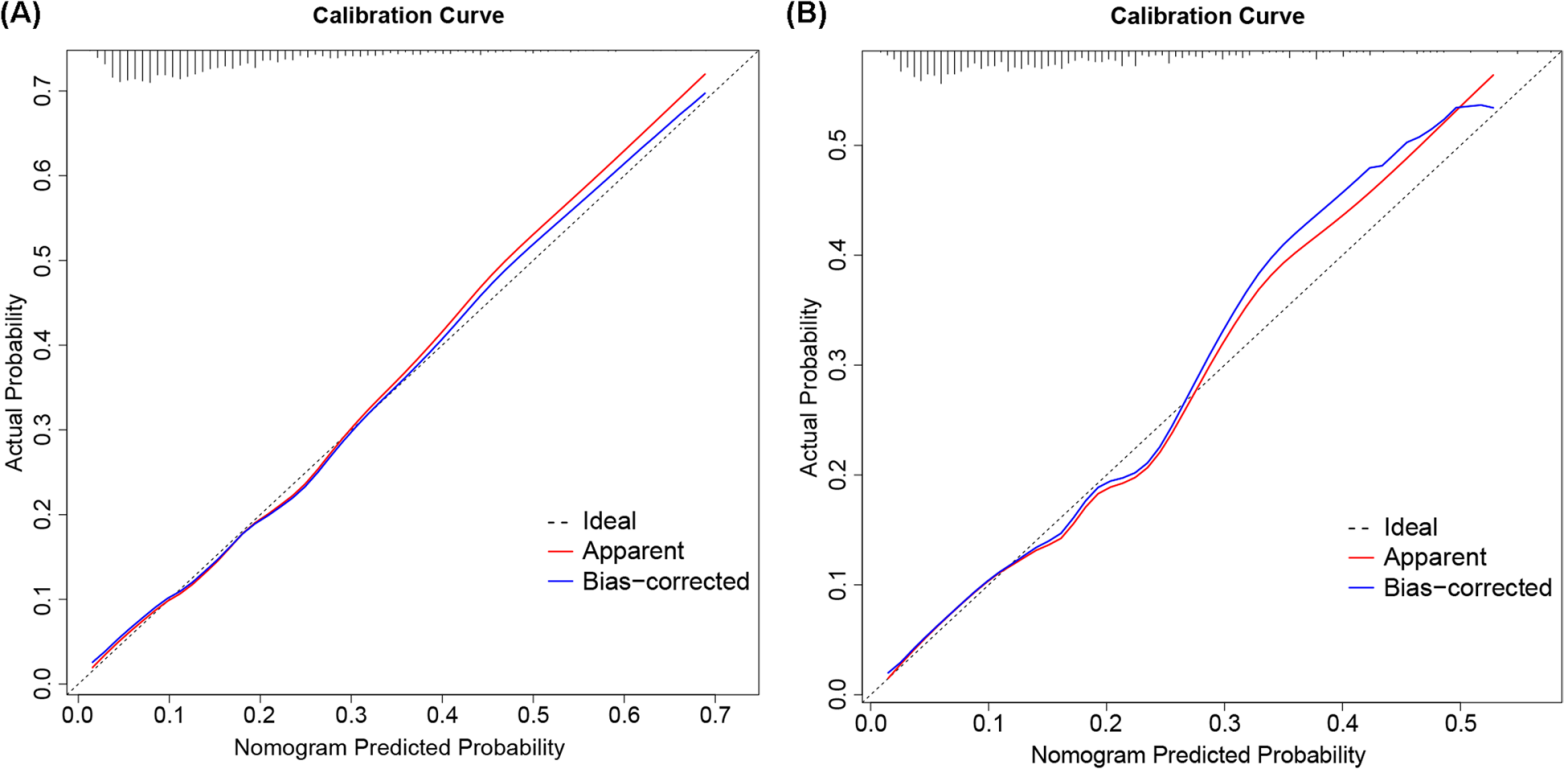
Calibration curves of the prediction nomogram in the training cohort (**A**) and validation cohort (**B**). The *x*-axis represents the nomogram-predicted probability, and the *y*-axis represents the actual probability of PAL. The black pointed line represents the ideal curve, the red solid line represents the apparent curve (non-correction), and the blue solid line represents the bias-correction curve by bootstrapping (B = 1000 repetitions). PAL, prolonged air leak

The discrimination capacity of the predictive model and nomogram was evaluated by the ROC curve (Fig. 3). The area under the ROC curve (AUC) was 0.7315 (95% CI: 0.6979–0.7651) for the training cohort and 0.7325 (95% CI: 0.6743–0.7906) for the validation cohort, indicating a relatively good prediction accuracy of the nomogram. The optimal cutoff value of the estimated probability of PAL was approximately 16%, and the sensitivity and specificity were 0.649 and 0.708 respectively (see Additional file 1). The Hosmer-Lemeshow test and calibration plot were used to assess the calibration power. The *P* value of the Hosmer-Lemeshow test was 0.388 for the training cohort and 0.577 for the validation cohort, which suggested an insignificant difference between the predicted probabilities and actual observed probabilities. The calibration plots for both the training (Fig. 4A) and the validation cohorts (Fig. 4B) also demonstrated a good calibration of the predictive nomogram. In addition, the predicted PAL probabilities of different risk categories based on the nomogram were closely consistent with the actual observed PAL rates in the validation cohort, as shown in Table 5.

**Table 5:** Risk categories of PAL in validation cohort

Risk categories	Predicted PAL risk (%)	Number of patients in validation cohort (n)	Incidence of PAL (***n***)	Observed frequency of PAL (%)
Low risk	< 10%	225	15	6.7
Intermediate risk	10%-20%	186	25	13.4
High risk	>20%	142	42	29.6

*PAL* prolonged air leak

### Clinical utility of the predictive nomogram

**Fig. 5:**
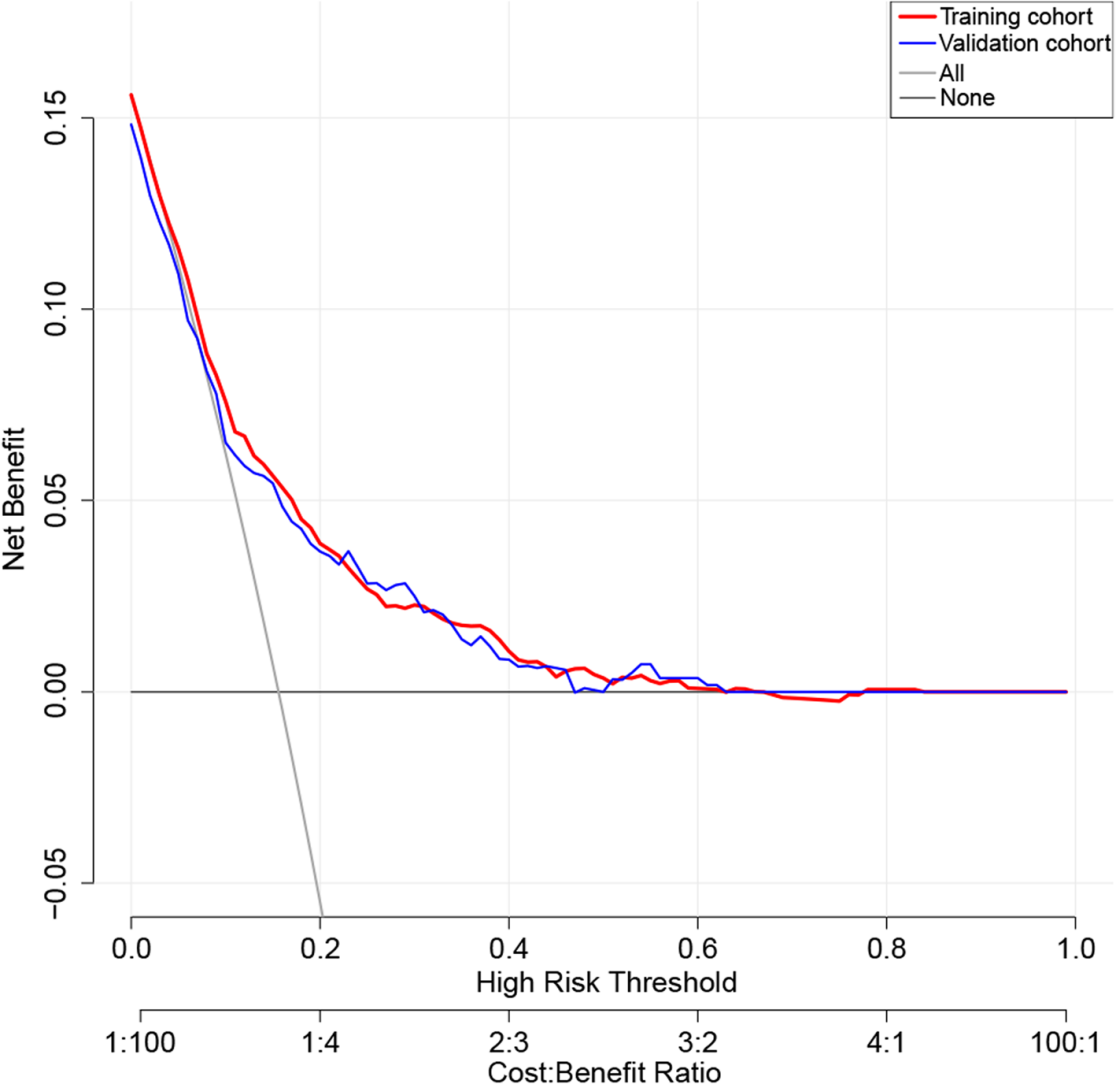
Decision curve analysis for the PAL nomogram in the training and validation cohorts. The *y*-axis measures the net benefit, the black line represents the assumption of PAL-none-patients, the gray line represents the assumption of PAL-all-patients, the red line represents the training cohort, and the blue line represents the validation cohort. PAL, prolonged air leak

DCA was applied to evaluate the clinical utility of the predictive nomogram (Fig. 5). The results showed that the nomogram provided a greater net benefit with a wider range of threshold probabilities for predicting the risk of PAL in both the training and the validation cohorts, suggesting that the nomogram was clinically useful and could enable surgeons to make better clinical decisions.

## Discussion

PAL remains one of the most frequent complications after lung surgery, with a reported incidence of approximately 5% to 30% [4–6]. Although minimally invasive surgical techniques have been relatively developed, PAL is still common, leading to a longer hospital stay, increased financial cost, and higher risk of postoperative infection [6, 8, 9]. In this study, we developed a clinical prediction model and devised a nomogram with good predictive performance for PAL after minimally invasive pulmonary resection. The probability of PAL for individual patients could be estimated using this predictive nomogram, and preventive measures could be adopted in advance for high-risk patients.

Prolonged parenchymal air leakage is often caused by impaired healing of damaged alveoli, which is usually associated with poor lung alignment with the parietal pleura [23]. In recent years, several effective innovations in techniques to deal with PAL intraoperatively and postoperatively have emerged, including fibrin sealants, pleural tents, water seal or suction, doxycycline pleurodesis, digital chest drainage system, and endobronchial valves [4, 11, 24–28]. Although these procedures can reduce the incidence of postoperative PAL, the routine use of these adjuncts might result in an increase of unnecessary cost and the waste of medical resources [29]. Therefore, a clinical prediction tool that can identify those patients with a high risk of PAL might guide the doctors to selectively use these adjuncts.

A large number of risk factors for PAL after lung surgery have been identified by prior studies [5–7, 30]. However, no studies have focused on the risk factors for PAL after minimally invasive pulmonary resection. Consistent with previous risk factor studies, we identified five independent risk factors for PAL: advanced age, low BMI, low FEV1% predicted, lobectomy, and smoking history. Age is an important factor affecting physical condition of the patients. Elderly patients usually have poor wound healing ability, and thus they are more prone to PAL [14, 31]. Overweight and obese patients are less likely to develop PAL due to their better nutritional status and intrathoracic milieu that favors sealing of lung parenchymal defects [12, 32]. Low FEV1% predicted and smoking history are closely associated with poor lung function, including reduced lung compliance and increased airway resistance, which could impair the recovering from lung injury [14, 33]. It is worth noting that we found that patients who underwent multi-lobar pulmonary resection were more likely to develop PAL. The lower level of pleural pressure caused by less resection of lung parenchyma during sublobar or mono-lobar resection might lead to a reduced risk of PAL [34]. The pleura is usually supported by the chest wall and by adhesions to the chest wall, thereby relieving pleural stress [35]. There will be less support and adhesion of the chest wall to the pleura if more lung parenchyma is removed, and the pleural pressure will increase correspondingly, thereby increasing the risk of PAL. Unlike the previous study performed by Attaar et al, we found that patients undergoing left-sided surgery were at higher risk of PAL compared with right-sided surgery. This might be due to the longer and hypoplastic oblique fissure of the left lung, resulting in greater damage to the pulmonary parenchyma during surgery [36, 37]. Interestingly, we first found that prolonged operation duration was an independent risk factor for PAL. Longer operation duration often means greater difficulty in surgery and more damage to lung tissue, which might increase the risk of PAL. In addition, the duration of lobectomy and multi-lobar pulmonary resection tends to be longer.

Although several models for predicting PAL have been reported previously [12–18], no model has been focused on predicting the risk of PAL after minimally invasive pulmonary resection. Compared with previously published predictive models for PAL, ours has the following advantages. First, we visualized this predictive model as a nomogram and then built an operation interface for our nomogram on the web page (https://lirongyangql.shinyapps.io/PAL_DynNom/), which greatly optimized the calculation process and improved the clinical usability of this model [38]. Second, we did not include patients undergoing thoracotomy and constructed a risk prediction model for predicting PAL after minimally invasive pulmonary resection for the first time. In an era of minimally invasive pulmonary resection as the mainstream surgical procedure, the clinical utility of our model might be better. Third, we developed a clinical model to predict the risk of PAL using preoperative and intraoperative characteristics without taking the patient's histopathological information into account. Although the pathological type and stage might be associated with the incidence of postoperative PAL [5], the histopathological results are usually not available prior to surgery and PAL often already occurs by the time we obtain the pathological results after surgery. With this model, we could identify high-risk patients at the end of surgery and intervene as early as possible to avoid PAL. Fourth, we investigated some intraoperative characteristics that have rarely been explored in previous studies but might be closely related to PAL (such as operation duration, number of LN dissected, and tumor size), and incorporated operation duration into the final model. In addition, unlike previous studies, we did not convert continuous variables (such as age, BMI, FEV1% predicted, and operation duration) to categorical variables, which provided our model with much greater discrimination accuracy between patients. Finally, we performed DCA to evaluate the clinical utility of the predictive nomogram [22]. The decision curve demonstrated an obvious net benefit of the nomogram model within the threshold range of 5–30% (incidence of PAL reported in prior literature), indicating a great clinical utility of this nomogram.

The clinical prediction nomogram constructed in this study could assist thoracic surgeons in assessing the risk of PAL in patients after minimally invasive pulmonary resection using preoperative and intraoperative characteristics and then make better clinical decisions. Preventive interventions could be applied to high-risk patients to reduce the incidence of PAL, while the potential harm and increased costs caused by overtreatment could be avoided for low-risk patients. For example, if the individual is identified as a high-risk patient at the end of the surgery, fibrin sealants or pleural tents could be immediately used to reduce the incidence of PAL. And digital chest drainage system and endobronchial valves could also be applied as early as possible to high-risk patients after surgery to avoid PAL. In our institution, we usually use the digital chest drainage system for high-risk patients or patients with suspected PAL to avoid the occurrence of PAL, which has achieved satisfactory outcomes. In addition, better preoperative patient counseling could be also achieved by informing high-risk patients of the possibility of stricter chest tube management, longer hospital length of stay, and higher hospitalization costs.

This study has several limitations that should be considered. First, the single-center retrospective nature of this study might limit the generalization ability of our predictive nomogram, and some uncontrolled confounders might also arise. In addition, the predictive model was only internally validated, thus the selection biases present in the training cohort might also be present in the validation cohort. Further external validation in a multicenter setting is required to determine whether this nomogram could be widely used in other populations. Finally, some factors that may be associated with PAL were not considered, such as pleural adhesion, diffusing capacity of carbon monoxide, surgeons’ experience and radiographic imaging characteristics, which were not available in our database and could be investigated in future research. Despite the above limitations, the independent risk factors for PAL identified in this study and the predictive nomogram constructed in this study could provide the reference for thoracic surgeons' clinical decision-making and pave the way for future research in this field.

## Conclusion

We developed a clinical nomogram for the prediction of PAL after minimally invasive pulmonary resection based on preoperative and intraoperative characteristics, and the nomogram achieved good predictive performance for PAL. The risk of PAL for individual patients can be estimated by using this nomogram, and preventive measures may be adopted in advance for those high-risk patients.

## Supplementary Information

**Additional file 1: Supplementary Table 1.** Results of ROC curve for training cohort sorted by Youden index (top 15).

## Data Availability

The data that support the findings of this study are available on request from the corresponding author.

## Abbreviations

PAL: Prolonged air leak
RATS: Robotic-assisted thoracic surgery
VATS: Video-assisted thoracic surgery
BMI: Body mass index
FEV1% predicted: Percentage of predicted value for forced expiratory volume in 1 second
MVV% predicted: Percentage of predicted value of maximal voluntary ventilation
COPD: Chronic obstructive pulmonary diseases
ASA: American Society of Anesthesiologists
PNI: Prognostic nutritional index
LN: Lymph node
ROC: Receiver operating characteristic
AUC: Area under ROC curve
DCA: Decision curve analysis
IQR: Interquartile range
SD: Standard deviation
CI: Confidence interval
OR: Odds ratio

## References

[1] Oudkerk M, Liu S, Heuvelmans MA, Walter JE, Field JK (2021). Lung cancer LDCT screening and mortality reduction - evidence, pitfalls and future perspectives. Nat Rev Clin Oncol.

[2] Demmy TL, Yendamuri S, D'Amico TA, Burfeind WR (2018). Oncologic equivalence of minimally invasive lobectomy: the scientific and practical arguments. Ann Thorac Surg.

[3] Shen H, Wang X, Nie Y, Zhang K, Wei Z, Yang F (2021). Minimally invasive surgery versus thoracotomy for resectable stage II and III non-small-cell lung cancers: a systematic review and meta-analysis. Eur J Cardio-Thor Surg.

[4] Bronstein ME, Koo DC, Weigel TL (2019). Management of air leaks post-surgical lung resection. Ann Transl Med.

[5] Hoeijmakers F, Hartemink KJ, Verhagen AF, Steup WH, Marra E, Roell WFB (2021). Variation in incidence, prevention and treatment of persistent air leak after lung cancer surgery. Eur J Cardio-Thor Surg.

[6] Liang S, Ivanovic J, Gilbert S, Maziak DE, Shamji FM, Sundaresan RS (2013). Quantifying the incidence and impact of postoperative prolonged alveolar air leak after pulmonary resection. J Thorac Cardiovasc Surg.

[7] Okereke I, Murthy SC, Alster JM, Blackstone EH, Rice TW (2005). Characterization and importance of air leak after lobectomy. Ann Thorac Surg.

[8] Varela G, Jiménez MF, Novoa N, Aranda JL (2005). Estimating hospital costs attributable to prolonged air leak in pulmonary lobectomy. Eur J Cardio-Thor Surg.

[9] Zhao K, Mei J, Xia C, Hu B, Li H, Li W (2017). Prolonged air leak after video-assisted thoracic surgery lung cancer resection: risk factors and its effect on postoperative clinical recovery. J Thor Dis.

[10] Dugan KC, Laxmanan B, Murgu S, Hogarth DK (2017). Management of Persistent air Leaks. Chest..

[11] Liberman M, Muzikansky A, Wright CD, Wain JC, Donahue DM, Allan JS (2010). Incidence and risk factors of persistent air leak after major pulmonary resection and use of chemical pleurodesis. Ann Thorac Surg.

[12] Attaar A, Winger DG, Luketich JD, Schuchert MJ, Sarkaria IS, Christie NA (2017). A clinical prediction model for prolonged air leak after pulmonary resection. J Thorac Cardiovasc Surg.

[13] Rivera C, Bernard A, Falcoz PE, Thomas P, Schmidt A, Benard S (2011). Characterization and prediction of prolonged air leak after pulmonary resection: a nationwide study setting up the index of prolonged air leak. Ann Thorac Surg.

[14] Brunelli A, Varela G, Refai M, Jimenez MF, Pompili C, Sabbatini A (2010). A scoring system to predict the risk of prolonged air leak after lobectomy. Ann Thorac Surg.

[15] Jin R, Zheng Y, Gao T, Zhang Y, Wang B, Hang J (2021). A nomogram for preoperative prediction of prolonged air leak after pulmonary malignancy resection. Transl Lung Cancer Res.

[16] Seder CW, Basu S, Ramsay T, Rocco G, Blackmon S, Liptay MJ (2019). A prolonged air leak score for lung cancer resection: an analysis of the Society of Thoracic Surgeons general thoracic surgery database. Ann Thorac Surg.

[17] Lee L, Hanley SC, Robineau C, Sirois C, Mulder DS, Ferri LE (2011). Estimating the risk of prolonged air leak after pulmonary resection using a simple scoring system. J Am Coll Surg.

[18] Pompili C, Falcoz PE, Salati M, Szanto Z, Brunelli A (2017). A risk score to predict the incidence of prolonged air leak after video-assisted thoracoscopic lobectomy: an analysis from the European Society of Thoracic Surgeons database. J Thorac Cardiovasc Surg.

[19] Obuchowski NA, Bullen JA (2018). Receiver operating characteristic (ROC) curves: review of methods with applications in diagnostic medicine. Phys Med Biol.

[20] Nattino G, Pennell ML, Lemeshow S (2020). Assessing the goodness of fit of logistic regression models in large samples: a modification of the Hosmer-Lemeshow test. Biometrics..

[21] Rosenfeld JP, Donchin E (2015). Resampling (bootstrapping) the mean: a definite do. Psychophysiology..

[22] Vickers AJ, Holland F (2021). Decision curve analysis to evaluate the clinical benefit of prediction models. Spine J.

[23] Sakata KK, Reisenauer JS, Kern RM, Mullon JJ (2018). Persistent air leak - review. Respir Med.

[24] Cordovilla R, Torracchi AM, Novoa N, Jiménez M, Aranda JL, Varela G (2015). Endobronchial valves in the treatment of persistent air leak, an alternative to surgery. Arch Bronconeumol.

[25] Mayor JM, Lazarus DR, Casal RF, Omer S, Preventza O, Simpson K (2018). Air leak management program with digital drainage reduces length of stay after lobectomy. Ann Thorac Surg.

[26] Hallifax RJ, Yousuf A, Jones HE, Corcoran JP, Psallidas I, Rahman NM (2017). Effectiveness of chemical pleurodesis in spontaneous pneumothorax recurrence prevention: a systematic review. Thorax..

[27] Allama AM (2010). Pleural tent for decreasing air leak following upper lobectomy: a prospective randomised trial. Eur J Cardio-Thor Surg.

[28] Malapert G, Hanna HA, Pages PB, Bernard A (2010). Surgical sealant for the prevention of prolonged air leak after lung resection: meta-analysis. Ann Thorac Surg.

[29] Singhal S, Ferraris VA, Bridges CR, Clough ER, Mitchell JD, Fernando HC (2010). Management of alveolar air leaks after pulmonary resection. Ann Thorac Surg.

[30] Cerfolio RJ, Bass CS, Pask AH, Katholi CR (2002). Predictors and treatment of persistent air leaks. Ann Thorac Surg.

[31] Petrella F, Rizzo S, Radice D, Borri A, Galetta D, Gasparri R (2011). Predicting prolonged air leak after standard pulmonary lobectomy: computed tomography assessment and risk factors stratification. Surgeon.

[32] Littleton SW (2012). Impact of obesity on respiratory function. Respirology (Carlton, Vic).

[33] Bluman LG, Mosca L, Newman N, Simon DG (1998). Preoperative smoking habits and postoperative pulmonary complications. Chest..

[34] Casha AR, Bertolaccini L, Camilleri L, Manche A, Gauci M, Melikyan G (2018). Pathophysiological mechanism of post-lobectomy air leaks. J Thoracic Dis.

[35] Casha AR, Manché A, Gatt R, Wolak W, Dudek K, Gauci M (2014). Is there a biomechanical cause for spontaneous pneumothorax?. Eur J Cardio-thoracic Surg.

[36] Garner JL, Desai SR (2021). Lung Fissural integrity: It's written in the genes. Am J Respir Crit Care Med.

[37] Bayter PA, Lee GM, Grage RA, Walker CM, Suster DI, Greene RE (2021). Accessory and incomplete lung fissures: clinical and Histopathologic implications. J Thorac Imaging.

[38] Bonnett LJ, Snell KIE, Collins GS, Riley RD (2019). Guide to presenting clinical prediction models for use in clinical settings. BMJ (Clinical research ed).

