# Supplementary material for: Construction and validation of a nomogram for predicting prolonged air leak after minimally invasive pulmonary resection
Source: World J Surg Oncol. 2022 Aug 3;20:249. doi: 10.1186/s12957-022-02716-w (PMC9347096; doi:10.1186/s12957-022-02716-w)
Supplement: Supplementary file 1 — Additional file 1: Supplementary Table 1. Results of ROC curve for training cohort sorted by Youden index (top 15). [file 12957_2022_2716_MOESM1_ESM.docx]

| **Sensitivity** | **1- specificity** | **Youden index** | **Predicted probability (%)** |
| --- | --- | --- | --- |
| 0.649 | 0.292 | 0.357 | 16.4 |
| 0.641 | 0.285 | 0.356 | 16.7 |
| 0.649 | 0.293 | 0.356 | 16.4 |
| 0.629 | 0.273 | 0.356 | 17.1 |
| 0.641 | 0.286 | 0.355 | 16.6 |
| 0.649 | 0.293 | 0.355 | 16.4 |
| 0.629 | 0.274 | 0.355 | 17.1 |
| 0.641 | 0.286 | 0.355 | 16.6 |
| 0.649 | 0.294 | 0.355 | 16.4 |
| 0.629 | 0.275 | 0.355 | 17.0 |
| 0.664 | 0.310 | 0.354 | 16.0 |
| 0.660 | 0.306 | 0.354 | 16.1 |
| 0.641 | 0.287 | 0.354 | 16.6 |
| 0.649 | 0.295 | 0.354 | 16.4 |
| 0.629 | 0.276 | 0.354 | 17.0 |

**Supplementary Table 1.** Results of ROC curve for training cohort sorted by Youden index (top 15).

ROC, receiver operating characteristic.
